# Injection of Affibodies by a Self-Organizing Bacterial Syringe to Interfere with Intracellular Signaling

**DOI:** 10.3390/toxins17090448

**Published:** 2025-09-05

**Authors:** Thomas Müller, Sophie Gieß, Fanny Maier, Lara Hofacker, Luca Stenger, Larissa Parker, Robert Grosse, Gudula Schmidt

**Affiliations:** Institute for Experimental and Clinical Pharmacology and Toxicology, Faculty of Medicine, University of Freiburg, Albertstr. 25, 79104 Freiburg, Germanyluca.stenger@email.uni-freiburg.de (L.S.);

**Keywords:** *Photorhabdus* toxin complex (PTC), affibody, Raf, signaling

## Abstract

*Photorhabdus luminescens* produces a syringe-like toxin complex to inject toxic enzymes into cells. We demonstrated that the recombinant *Photorhabdus* toxin complex (PTC) can be engineered for translocation of foreign cargo proteins across cellular membranes. We showed that the system is suitable for injection of trimeric affibodies into mammalian cells in order to influence crucial signaling pathways. As proof of principle, we inhibited Ras-driven tumor cell proliferation by injection of an affibody which interacts with the Ras binding domain of Raf kinase. The system described here could be applicable to target a wide range of signaling molecules for cell biological or therapeutic intervention.

## 1. Introduction

Biological drugs account for about half of the new approvals by the United States Food and Drug Administration (FDA) in the last five years [1]. Moreover, proteins and peptides are frequently used for cell biological studies. Protein-based drugs or tools like hormones, growth factors, enzymes, monoclonal antibodies, and antibody–drug conjugates show good target specificity. Nevertheless, one of the major drawbacks of most peptide-based drugs and tools is their inability to effectively cross the plasma membrane and reach potential targets within the cell [2]. Exploring ways and means for protein-based therapeutics and tools to bypass the phospholipid bilayer is therefore of great importance to the advancement of the field. Bacterial toxins provide a naturally evolved delivery system capable of transporting functional proteins into mammalian cells [3].

Pathogenic bacteria produce and release exotoxins to modulate the eukaryotic host environment for their benefit. These bacterial toxins act as autonomous molecular devices which modify intracellular target proteins or perforate cellular membranes to cause cell death.

*Photorhabdus luminescens* is a nematode-associated insect pathogen which produces a syringe-like toxin complex to inject enzymes into insect cells. Such bacterial toxin complexes (Tc toxins) are also produced by other *Photorhabdus* and *Xenorhabdus* species [4,5]. Similar in function is the injection system produced by *Photorhabdus asymbiotica* (PVC) identified recently [6,7]. All these machines are solely formed by proteins and do not require living bacteria to be active, as is the case for type-III-secretion [8]. The *Photorhabdus* toxin complex (PTC) is composed of three subunits: TcA, TcB, and TcC [9]. These come together in a 5:1:1 (A:B:C) stoichiometry and shape a high molecular mass (~1.7 MDa) tripartite ABC-type holotoxin [10]. So far, seven TcA-type, three TcB-type and seven TcC-type isoforms have been identified in the *Photorhabdus luminescens* strain TTO1. These are able to form biologically active PTCs in different combinations [10,11]. Oligomerization of TcA to a homo-pentamer results in the formation of a large (~1.4 MDa) structure that contains a central syringe-like channel surrounded by a shell [12]. This toxin part facilitates cell binding and injection of Tc toxins. TcB and TcC congregate into a cocoon-like complex that interacts with the top of the TcA injection channel thereby completing the holotoxin [13,14]. The enzymatic part of PTCs is located within the so-called hypervariable region (hvr) at the C-terminus of each TcC [14,15]. The payload is autoproteolytically separated from the TcC N-terminus by the *Photorhabdus* metalloprotease PrtA1 during BC-complex assembly [10].

Injection is triggered by pH changes. The basic nature of the insect gut or acidification of maturing endosomes in mammalian cells likewise induces conformational changes within the TcA pentamer. These include a concerted constriction of alpha helices which connect the “needle” with the “shell” allowing perforation of biological membranes in a syringe-like fashion [16]. The catalytically active C-terminal hvr of TcC is translocated through the TcA pore into the cytosol [17,18,19] (compare the summarizing figure at the end of this manuscript).

It has been demonstrated that PTC, which is composed of the isoforms TcdA1, TcdB1, and TccC3, can be engineered into an injection nanomachine suitable for translocation of foreign proteins [20,21]. TcdB1 and TccC3 can be functionally expressed as one fusion protein, designated as BC throughout this document. Recombinant replacement of the TcC-hvr with other proteins allowed their injection into living cells. Examples of successful injection are C3bot toxin from *Clostridium botulinum*, a modified version of the YopT effector from *Yersinia enterocolitica* or MLuc7 luciferase from the copepod *Metridia longa* [20]. The restrictions for PTC-translocated cargos are not fully understood yet. However, successful delivery of foreign proteins seems to require them having a PI ≥ 8 and a size of naturally occurring TcC-hvrs (about 35 kDa) [20]. Although there might be more, yet unknown prerequisites, the list of suitable peptides fitting these criteria is long.

Affibodies are resilient scaffold proteins derived from the IgG-binding Z-domain of staphylococcal protein A [22]. Structurally, affibodies are composed of 58 amino acids (~6.5 kDa) that are organized in a bundle formed of three α-helices [23]. Therefore, affibodies are not structurally related to immunoglobulins. However, selected amino acids in affibodies can be replaced. This changes the original binding properties with new affinities to any target protein of choice. First affibody libraries and subsequent selection strategies for molecular targeting were reported about 30 years ago [24,25]. Such libraries are typically based on the randomization of 13 outward-facing amino acids within helices one and two (H1/H2) of the three-helix Z-domain scaffold [26,27]. More recent secondary library generation and selection have produced affibody molecules with high affinities to their targets (in the picomolar range) [28,29,30]. Meanwhile, affibodies directed against several different target proteins have been isolated and used in over 400 studies, with more being added to the list every year [31]. For example, affibody-based tracer molecules have been developed against several cancer-related targets [32,33,34,35]. Preclinical studies indicate the potential of affibodies as possible alternatives to therapeutic antibodies. Affibodies modulate signaling of insulin-like growth factor 1 receptor (IGF1-R), platelet derived growth factor receptor-beta (PDFRβ), human epidermal growth factor receptor 2 and 3 (HER2, HER3), and vascular endothelial growth factor receptor 2 (VEGFR2) [36,37,38,39].

Potential efficacy is based on the extracellular disruption of ligand–receptor interactions. Affibodies show much better tissue penetration, extravasation, and tumor penetration compared to antibodies [40]. However, an essential part of signal transduction takes place within the cell and is facilitated by a large array of downstream signaling components like small GTPases, kinases, and transcription factors. Recently, an affibody targeting an important intracellular signaling pathway has been generated. It binds with high affinity to the Ras binding domain of the serine/threonine-protein kinase Raf (affibody Zraf320) [41,42]. Inhibitory effects of this affibody on TNFα-induced signaling has been demonstrated in MH7A synoviocytes [43]. In these studies, delivery of this protein into the cytosol was achieved via plasmid-based transient expression or by protein transfection utilizing cell-penetrating peptides. In comparison to antibodies, affibodies are small molecules and lack secondary modifications. This fact opens up the possibility of membrane translocation via less disruptive delivery systems such as bacterial AB-toxins. Successful translocation of biologically active affibodies into mammalian cells has been reported utilizing the anthrax toxin PA pore in combination with an LF_N_-fused Raf-targeting affibody [44].

The development of protein translocation methods is of great interest not only to the affibody field, but to all research aiming at intracellular applicability of peptide-based therapeutics. In this study, we explore the potential of the *Photorhabdus* toxin complex to inject biologically active affibodies into the cytosol of mammalian cells. As proof of principle, we delivered the wildtype Z-domain scaffold, as well as the Raf-targeting affibody by PTC into mammalian cells to prove functional interference of the later with downstream signaling.

## 2. Results

### 2.1. Injection of an Affibody–Toxin Fusion into Mammalian Cells

**Figure 1:**
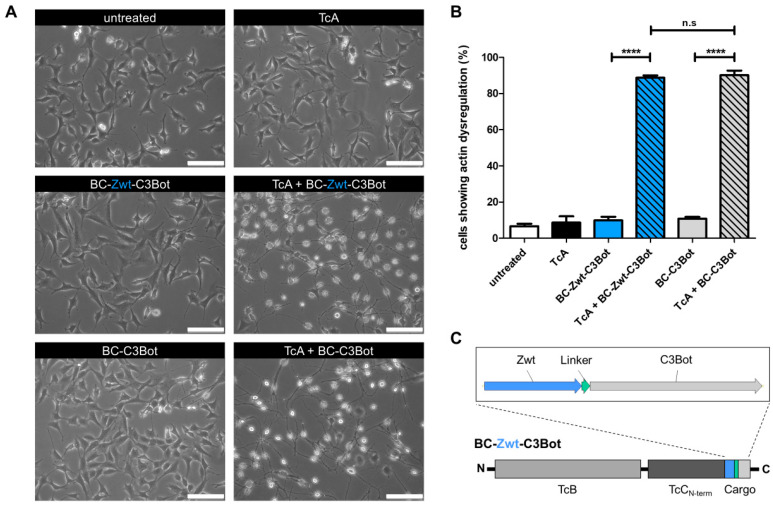
An affibody–toxin fusion as cargo can be injected into mammalian cells. (**A**) Representative bright field images of MEF cells treated with either TcA, TcB-TcC (BC) fusion protein, or PTC holotoxin containing either Zwt-C3Bot or C3Bot as cargo. Cells were intoxicated overnight at 37 °C. TcA was used at a concentration of 2 µg/mL, and BC variants at 1 µg/mL. (**B**) Quantification of bright field images. Morphologically normal and rounded MEF cells were counted across different treatment groups over the course of three independent experiments (N = 3). Statistical analysis was performed using ANOVA with Bonferroni’s multiple comparison test (n.s., not significant; **** *p* < 0.0001). (**C**) Schematic representation of the TcB-TcC (BC) fusion protein containing Zwt-C3Bot as cargo. The Zwt affibody (blue) is positioned upstream of the cargo sequence and connected to the C3Bot toxin via a linker (teal).

It has been shown that PTC can be reshaped into molecular injection machinery by replacing the wildtype TcC-*hvr* with foreign protein cargos [20,21]. Several cargo proteins can be injected by the *Photorhabdus luminescens* toxin machinery (PTC) into mammalian cells, including ADP-ribosyl transferases, proteases, or a luciferase [20]. Affibodies, which are small and variable affinity proteins, are interesting candidates for translocation into the cytosol via this bacterial toxin complex. Here, we tested whether affibodies may be injected to influence intracellular signaling pathways. Affibodies are composed of three α-helices with high structural stability and the property to refold following de-arrangement. Therefore, we asked whether PTC would be able to inject an affibody into mammalian cells. For easier detection, we generated a new BC subunit in which the hvr was exchanged for a fusion of the wildtype affibody (Zwt) and C3Bot of *Clostridoides botulinum* (BC-Zwt-C3Bot), as depicted in Figure 1. The fusion protein has a size of 30.4 kDa and a basic PI (9.25). C3Bot is a bacterial toxin which modifies RhoA by ADP ribosylation. Successful injection of this affibody–toxin fusion into cells by PTC should lead to rapid inactivation of RhoA. Subsequent destruction of the cytoskeleton should induce visible cell rounding. As positive control for functional injection, we used PTC-C3Bot composed of the A component and BC-C3Bot. As shown in Figure 1A, treatment of MEF cells with PTC-C3Bot (TcA + BC-C3Bot) or with PTC-Zwt-C3Bot (TcA + BC-Zwt-C3Bot) induced rounding of cells, indicating functional injection of both cargo proteins. In contrast, neither the A component alone nor the isolated BC-C3Bot or BC-Zwt-C3Bot induced cell rounding. Data are quantified in Figure 1B. Our data show that generally affibodies can be injected by PTC into mammalian cells.

### 2.2. Z3-Affibody Construction and Binding Studies

**Figure 2:**
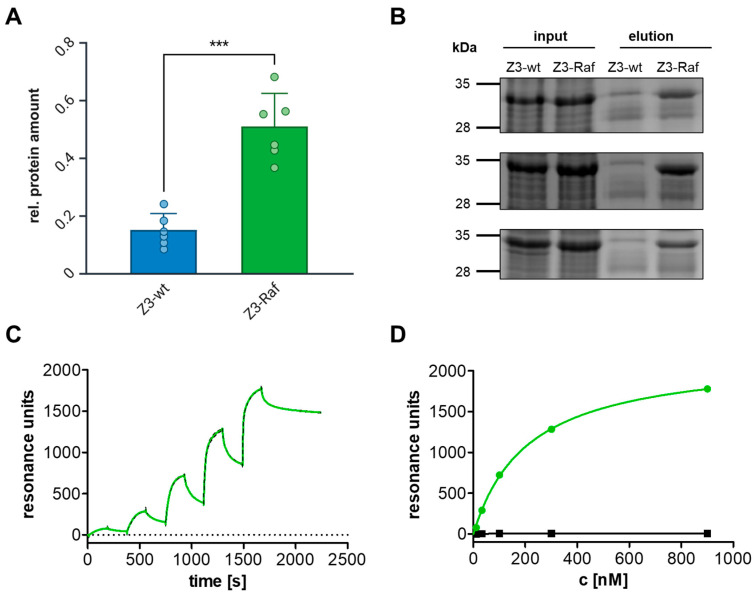
The trimeric affibody Z3-Raf binds to Raf with high affinity. (**A**) Pulldown assay of Z3-wt and Z3-Raf affibodies from the soluble fraction of bacterial lysates using GST-tagged C-Raf RBD coupled to a Sepharose solid phase. Data represent the quantification of six independent experiments (N = 6) and are shown as the mean ± standard deviation of the relative protein amount, calculated as the ratio between input and elution signals. Single data points are given, additionally. Statistical significance was determined using an unpaired Student’s *t*-test (***, *p* < 0.001). (**B**) Representative Coomassie-stained SDS-polyacrylamide gels showing Z3 affibody bands in the input and elution fractions of the pulldown experiment. Affibody proteins are indicated by arrows. (**C**) Surface plasmon resonance (SPR) analysis of C-Raf RBD (green) binding to immobilized Z3-Raf affibody on a CM5 sensor chip. In single-cycle measurements, RBD concentrations were sequentially increased from 11 nM to 900 nM (11 nM, 33 nM, 100 nM, 300 nM, and 900 nM). Each concentration included an association phase of 180 s, followed by a dissociation phase of 360 s. The graph represents the mean of three independent measurements (N = 3), with the striped line indicating the standard deviation (SD). Kinetic parameters were determined using a 1:1 binding model. (**D**) Steady-state dose–response curves of C-Raf RBD or CDC42 binding to immobilized Z3-Raf at equilibrium. Equilibrium binding curves were generated using a one-site specific binding model.

The previously described affibody Zraf322 targeting the Ras binding domain (RBD) of the serine/threonine-protein kinase Raf is a sensible example for testing whether injected affibodies influence signaling pathways within cells [41]. Therefore, we intended to exchange the toxins catalytic domain (TcC-hvr) for the wildtype or for the Raf affibody. However, a single affibody as cargo would be too small to be injected. Therefore, the first step to achieve injection of affibodies was to engineer cargo constructs that meet the translocation requirements known for PTC-injected cargos, namely molecular size from 16.5 kDa to 35 kDa and basic pI [20]. The following affibody constructs were chosen as best suitable for injection by PTC: three Zraf322 affibodies, as well as three times the wildtype Z-domain (Zwt) were chained together to homo-trimeric Z3-affibody constructs containing a C-terminal biotinylation tag (Avi tag) for detection. The proteins were named BC-Z3-wt (depicted in blue) and Z3-Raf (represented by green color) (Figure S1A,B). They match the size criteria of naturally occurring TcC-hvr and comply with the isoelectric point requirements for PTC-mediated translocation (pI >8) due to adding an N-terminal lysin loop. For expression and purification of BC-Z3-affibody fusion proteins, Z3-constructs were inserted into the pET-28a-BC vector (cargo, Figure S1C, left). Z3-affibodies were additionally cloned into the plasmid pcDNA3.1/His-C for eukaryotic expression. Following assembly of TcA with BC to PTC, the cargo is cleaved within the cocoon (Figure S1C, right). To block signaling within cells, affibodies have to be injected. Moreover, these engineered affibodies have to bind with high affinity and specificity to their target proteins. Therefore, the direct interaction between Z3-affibodies and Raf was analyzed. Zraf322 was directed against the Ras binding domain of Raf, which is conserved in all Raf isoforms. We performed a pulldown assay with Z3-wt and Z3-Raf affibodies from the soluble fraction of bacterial lysates using GST-tagged C-Raf RBD coupled to a Sepharose solid phase. The high affinity binding of Z3-Raf allowed us to pull Z3-Raf but not Z3-wt with beads-coupled recombinant Raf-RBD (Figure 2A,B). To characterize the binding kinetics between the trimeric affibodies and their respective target proteins, we utilized plasmon resonance (Biacore) measurements. The binding of a protein to its immobilized interaction partner results in a shift in the light reflection angle that can be monitored in real-time. Therefore, we immobilized the recombinant trimeric affibodies to dextran CM5 sensor chips and let recombinant C-Raf (green line) or Cdc42 (negative control, black line) run over the lane. We probed increasing concentrations (11 nM to 900 nM) of the target proteins. Each binding interval was followed by dissociation with buffer running over the lane. The trimeric Raf affibody binds with even higher affinity to its target as compared to the monomeric affibody (KD Zraf322: 1.9 µM, KD Z3-Raf: 25 nM). However, it shows no measurable affinity for the small GTPase Cdc42 (Figure 2C,D). The binding kinetics suggest a possible effect for Z3-Raf when injected into cells.

### 2.3. In Vitro Injection of Trimeric Affibodies

**Figure 3:**
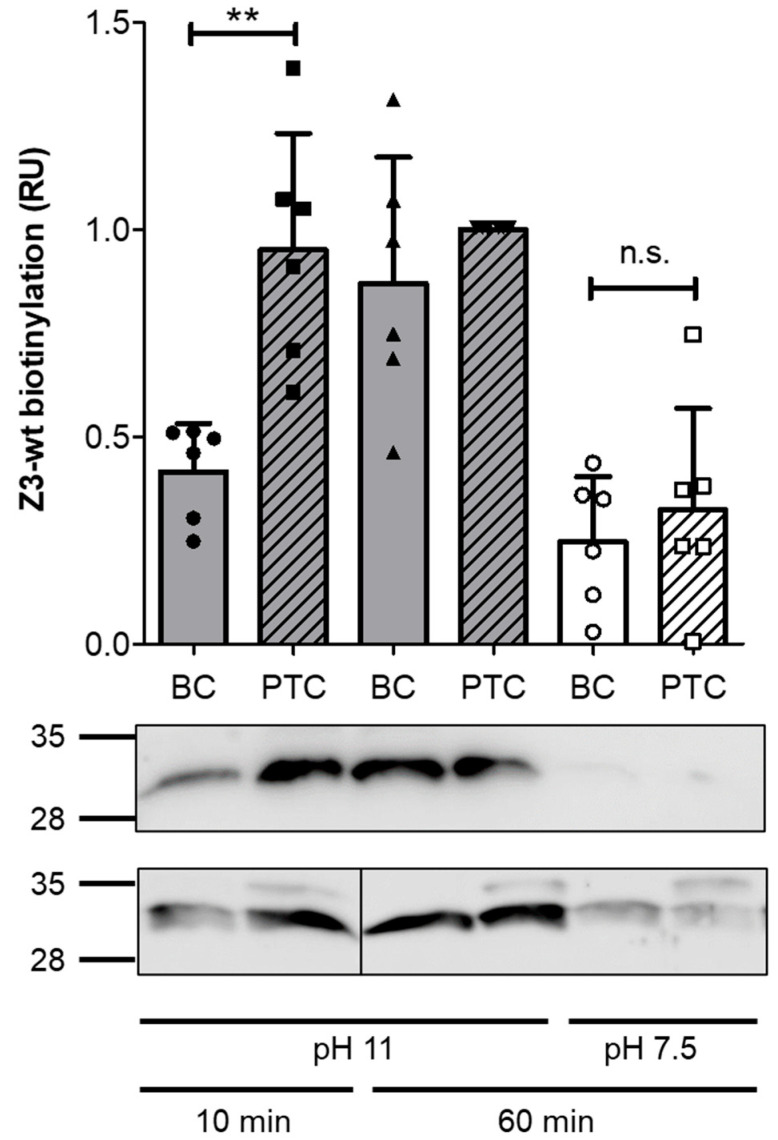
The trimeric affibody Z3-wt is released from the PTC by pH change. Western blot analysis of biotinylated Z3-wt affibody following ejection from the toxin complex. BC-Z3-wt was incubated either alone or with TcA to facilitate the assembly of the PTC holotoxin. Cargo ejection was triggered by a pH change to 11. Data represent the quantification of six independent experiments (N = 6), with signals normalized to the biotinylation levels of Z3-wt ejected from PTC at pH 11 after 60 min. Two independent experiments are shown. All data are presented as means ± standard deviation. Additionally, each single data point is shown. Statistical analysis was performed using one-way ANOVA with the Newman-Keuls multiple comparison test (** *p* < 0.01; n.s., not significant).

We asked whether trimeric affibodies are also suitable for injection through the narrow opening of the PTC syringe. Therefore, we performed in vitro injection experiments as described by Roderer et al. [21]. To this end, we incubated TcA and BC-Z3-wt for one hour on ice to allow formation of the complete PTC. In the assembled PTC, the cleaved trimeric affibody is already loaded from BC-Z3-wt into the needle [14]. Then, we changed the pH of the buffer to 11 or incubated the PTC at neutral pH (Figure 3). PH 11 represents the basic milieu in the gut of insect larvae. It induces conformational changes for injection of the cargo into the buffer. For detection of the isolated affibodies, we biotinylated the Avi tag of the free protein. The affibody enclosed inside the cocoon or the needle should not be accessible for the modifying enzyme. Biotinylated proteins were then detected by Western blotting (Figure 3). Our experiment shows that the in vitro injection is very fast. Within 10 min incubation at pH11, the affibody was efficiently released from the PTC. In contrast, incubation of BC-wt without TcA at pH 11 led to less biotinylation, suggesting that the affibodies are still mainly covered within the cocoon. pH-dependent destruction of the cocoon may explain accessibility of some affibodies. In line with this, following 60 min incubation at pH 11, the affibodies from the standalone BC-wt were biotinylated. Equal amounts of labeled affibodies appear in the Western blot. This indicates that this basic condition within 60 min leads to complete destruction of the BC-wt cocoon and to the release of the remaining affibodies. However, when incubated at neutral pH, affibodies from the isolated BC-wt cocoon and also of the complete PTC were stably covered within the cocoon and therefore not significantly biotinylated. The data indicate that it is generally possible to inject trimeric affibodies by PTC.

### 2.4. Effects of Z3-Affibody Expression on AP1 Activity

Previously, it was shown that transient expression of the monovalent Zraf322 affibody in synoviocytes leads to inhibition of TNFα-induced phosphorylation of ERK [43]. Similar results were obtained when the Raf targeting affibody was transported into cells via the anthrax PA/LF toxin [44].

**Figure 4:**
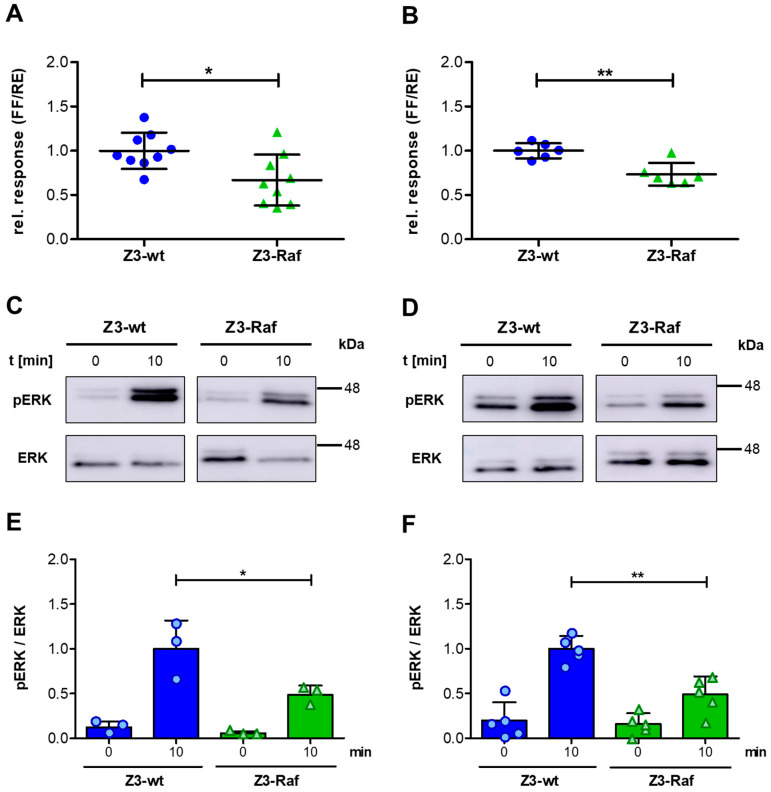
The injected affibody Z3-Raf is active in cells. (**A**) AP-1 luciferase reporter assay in HeLa- and (**B**) Panc-1 cells transiently transfected with pcDNA3.1 vectors encoding Z3-wt (blue, circles) or Z3-Raf (green, triangles) affibodies. Data are expressed as the ratio of firefly (FF) to Renilla (RE) luciferase activity, normalized to luminescence signals from Z3-wt transfections. Results are presented as mean ± standard deviation from nine independent experiments (HeLa) and six independent experiments (PANC). Statistical significance was determined using Student’s *t*-test (* *p* < 0.05; ** *p* < 0.01). (**C**) Immunodetection of ERK and phosphorylated ERK (pERK) in HeLa and (**D**) Panc-1 cell lysates before and after EGF stimulation (20 ng/mL), analyzed via Western blotting. Cells were serum-starved overnight and treated for 2 h with PTC containing Z3-wt or Z3-Raf affibodies as injectable cargos before EGF stimulation. (**E**) Quantification of ERK and pERK immunodetection in HeLa and (**F**) Panc-1 cells. Data are presented as the ratio of pERK to total ERK, normalized to lysates pretreated with PTC-Z3-wt and stimulated with EGF for 10 min. Results are presented as mean ± standard deviation, with N = 3 for HeLa cells and N = 5 for Panc-1 cells. Additionally, single data points are shown. Statistical significance was assessed using one-way ANOVA with the Bonferroni post hoc test (* *p* < 0.05; ** *p* < 0.01).

To elucidate whether the trimeric (Z3Raf)-affibody mediates comparable effects in mammalian cells, we studied its effect on the Ras-induced transcriptional activation. The transcription factor 1 (AP1) regulates gene synthesis downstream of the Ras-Raf-MAPK signaling pathway. AP1 activity was studied utilizing a dual-luciferase reporter assay. Therefore, HeLa cells were co-transfected with a reporter plasmid encoding AP1-controled firefly luciferase, a plasmid allowing constitutive expression of *Renilla* luciferase as transfection control and plasmids encoding each Z3-affibody (Z3-wt or Z3-Raf). Following transfection, cells were cultivated for further 16 h, lysed, and luciferase activity was measured. Under these conditions, expression of Z3-Raf affibodies resulted in significantly reduced luciferase production compared to the Z3-wt-expressing controls (Figure 4A). This indicates that the trimeric Raf affibody was able to inhibit serum-induced AP1 activation in HeLa cells.

The majority of pancreas carcinomas are based on dominant active p21 Ras mutations [45]. To study whether the affibodies would have a similar effect in cells expressing dominant active Ras, we repeated the same assay in pancreas carcinoma cells (Panc-1) which express a dominant active p21 Ras mutant. Again, expression of the Z3-Raf affibody reduced AP1-dependent synthesis of firefly luciferase compared to Z3-wt affibody expressing cells (Figure 4B). Our data indicate that expression of Z3-Raf affibodies was sufficient to inhibit the Ras-Raf signaling cascade in mammalian cells, even in the presence of dominant active Ras.

### 2.5. Effects of Z3-Affibody Injection on ERK Phosphorylation

We have shown previously, injection of cargos using the PTC is much more effective compared to transfection. We identified nearly 100% of cells to be injected [20]. To study whether the amount of Z3-affibodies injected by PTC may be sufficient to reduce AP1 activation, phosphorylation of ERK following stimulation of serum-starved cells with epidermal growth factor (EGF) was analyzed. Therefore, serum-starved cells were treated with PTC in an estimated amount of 10^8^ PTCs per cell. The Ras signaling cascade was stimulated 2 h later by addition of EGF (20 ng/mL) for 10 min or cells left untreated. Cells were lysed on ice, and the cytosolic fraction analyzed for phosphorylated ERK by Western blotting. Indeed, in HeLa cells incubated with PTC-Z3-Raf ERK phosphorylation was significantly lower than in cells incubated with PTC-Z3-wt (Figure 4C,E). The data show that the affibodies are readily injected by PTC into cells and that the injected affibodies seem to be correctly folded within cells to bind to their target proteins. We repeated this analysis using Panc-1 cells which express a dominant active Ras mutant. Again, injection of Z3-Raf robustly reduced EGF-stimulated ERK phosphorylation (Figure 4D,F). The data suggest that Z3-Raf acts as a reliable inhibitor of the Ras-Raf pathway even in cells expressing dominant active Ras. Altogether, our data indicate that incubation of mammalian cells with PTC-Z3 affibodies led to injection of the trimeric affibodies into the cytosol and that the amount of injected affibodies is sufficient to inhibit Ras–Raf signaling. Therefore, injection of affibodies into mammalian cells could be a new general method to selectively modulate the action of intracellular proteins in living cells.

### 2.6. Colony Formation and Growth Following Affibody Injection

**Figure 5:**
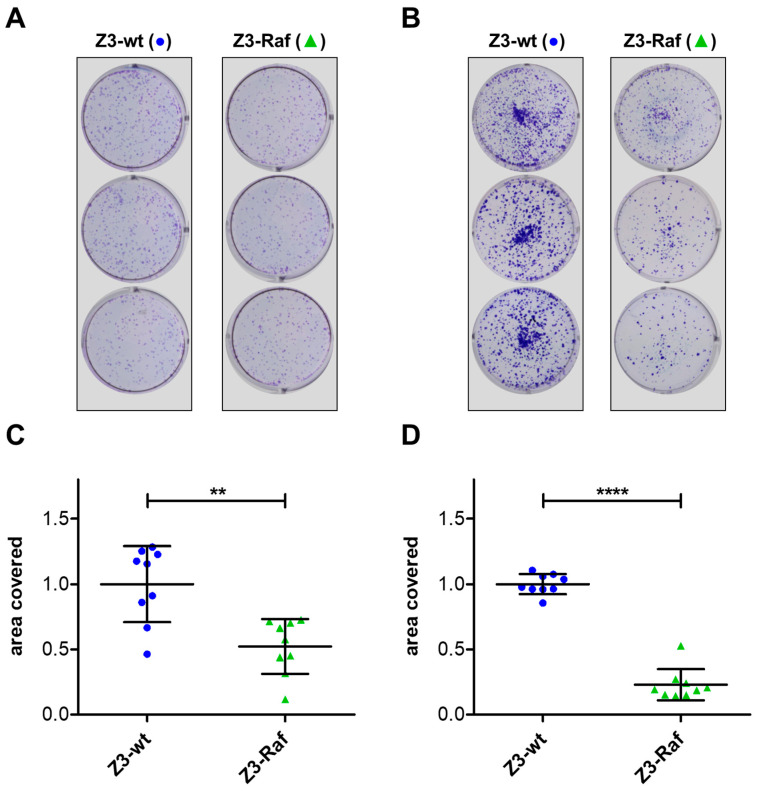
Long-term effect of the injected trimeric affibodies. Colony formation assay of HeLa and Panc-1 cells injected with Z3 affibodies via PTC. (**A**) Representative images of stained HeLa and (**B**) Panc-1 cell colonies grown from single cells over a 10-day period in 35 mm dishes. Cells were pretreated for 1 h with PTC containing either Z3-wt or Z3-Raf affibodies. Surface-bound PTC was triggered by lowering the medium pH. Afterward, cells were washed, detached, and re-seeded at equal numbers. (**C**) Quantification of colony formation by HeLa and (**D**) Panc-1 cells. Data are presented as the area covered by cell colonies for each group. The mean of three experiments on one day from PTC treatment with Z3-wt as cargo was normalized to 1. Mean values with standard deviations from all nine experiments (N = 9) are shown. Statistical analysis was performed using Student’s *t*-test (** *p* < 0.01; **** *p* < 0.0001).

Nothing is known about the long-term effects and the fate of injected affibodies within cells. We intended to perform an experiment analyzing the effect of injected affibodies for some days. To this end, colony formation assays were performed with HeLa, Panc-1, and Capan-2 cells which were exposed to PTC holotoxins harboring the three different Z3-affibodies, respectively. For an optimal injection rate, the cells were incubated with the PTCs for 60 min to allow for binding and endocytosis. Then, the medium was exchanged for a buffer with pH 5.8 to mimic the acidic pH of maturing endosomes. This pH change induces the direct injection of affibodies through the plasma membrane from PTC still on the cell surface and enhances injection efficacy [20]. Cells were detached from the plate and washed to remove all unbound PTCs. Equal numbers of cells of each group were seeded into six-well plates and grown for ten days in full medium. Grown colonies were fixed and stained (Figure 5A,B). As indicator for colony formation and growth, plates were then analyzed for the total area covered by cells using the ImageJ program (Figure 5C,D). Data were normalized for the results achieved with injection of the wildtype affibody. The total area covered with HeLa or Panc-1 cells was significantly reduced when cells were injected with Z3-Raf in relation to cells injected with Z3-wt. The experiments were repeated with Capan-2 pancreas carcinoma cells as an additional cell line with similar results (Figure S2).

**Figure 6:**
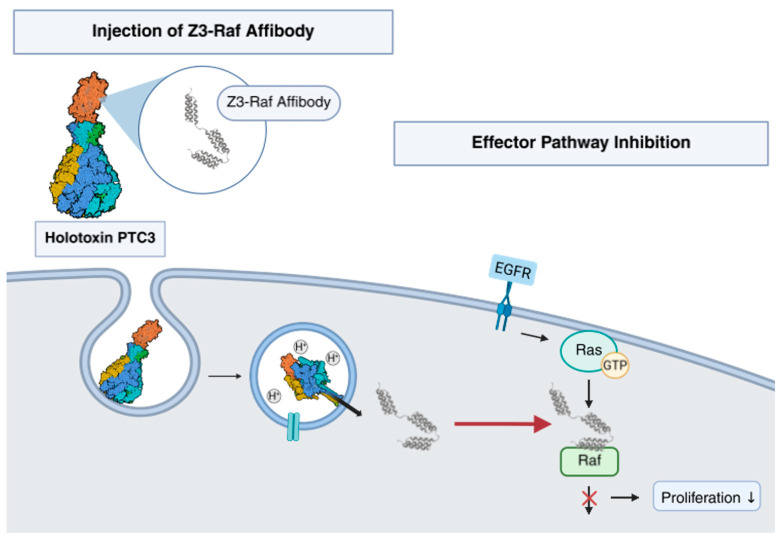
Model of PTC-mediated injection of affibodies.

Altogether, our data show that affibodies directed against intracellular proteins can be injected by PTC. Moreover, the amounts of cytosolic affibodies seem sufficient to interfere with the respective signaling function. Therefore, engineered PTC enables the controlled delivery of affibodies into mammalian cells. PTC-injected affibodies are an important addition to cell biological and probably futural pharmacological methods and tools (depicted in Figure 6).

The *Photorhabdus* toxin complex (PTC) forms a standalone injection machinery. It is composed of a pentameric A-component (multicolor) and a BC-component (red). BC encloses a cargo to be injected (here, a trimeric affibody). Following binding and endocytosis, the affibody is injected through the endosomal membrane into the cytosol of mammalian cells. There, it interacts with its target protein to block signaling (here, Ras–Raf signaling). The structure shown was adopted from [14]. 

## 3. Discussion

The growing number of antibodies and peptide-based drugs, which are used therapeutically, proves the potential of utilizing protein–protein interactions for modulating target cell behavior in cell biology, pharmacology, and medicine. However, functional therapeutic proteins are not readily taken up into the cytosol of mammalian cells. This fact prohibits the possibility to directly influence mis-controlled intracellular target molecules like oncogenic Ras- or Raf-variants. Delivery of recombinant proteins that interfere with intracellular signaling pathways is still a great challenge. Several translocation methods are in development, for example, liposomes, nanocarriers, viruses, cell-penetrating peptides, pore-forming bacterial toxins, and—as described here—injection by the *Photorhabdus* Toxin Complex (PTC). All methods have their own drawbacks, such as expression of proteins encoded by viral DNA/RNA or delivery of genes by liposomes lack sufficient control of peptide production levels. The transportation of peptides by pore-forming toxins has the disadvantage of instability within the host serum where peptides are quickly degraded by proteases and peptidases.

Direct translocation through the plasma membrane via amphiphilic molecules avoids endocytosis and thereby lysosomal cargo destruction. However, this method has limited release efficiency and the cargo size is restricted due to micellar pore formation (for review see [46]).

Recently, we and others demonstrated that the *Photorhabdus* toxin complex can be engineered into a non-toxic injection nanomachine, suitable for translocation of various cargo proteins into cells [20,21]. The machinery exhibits several key properties: First, the injection apparatus is exclusively formed by two recombinant proteins (TcA and the BC fusion protein) which can be purified from *Escherichia coli* and autonomously assembled when added to culture medium. Second, because recombinant proteins cannot expand, the maximal amount of injected cargo can be precisely controlled. Each PTC is able to inject only one protein into the cell it binds to, with no possibility of re-loading. Third, the protein intended to inject is hidden in a cocoon-like structure and thereby protected from rapid degradation by proteases and peptidases. Additionally, possible extracellular effects induced by the cargo are diminished due to shielding by the cocoon.

We previously demonstrated that the size and the isoelectric point (pI) of the cargo restrict injection by PTC; affibodies are small (6–8 kDa) and therefore could easily be adapted to these requirements. However, it is not known whether folded proteins can be pushed through the needle formed by the TcA pentamer. A recent publication analyzing prepore-to-pore transition suggests a tip diameter of about 7.5 Å, which would permit the passage of an alpha helix but not of a stable bundle of three helices the affibodies are composed of [47]. The native Z-domain of the wildtype affibody is expected to encompass the highest intramolecular stability. Therefore, we first studied if this affibody could be injected by the toxin complex. As an indicator for injection, we fused *Clostidoides botulinum* C3 toxin to the affibody scaffold and investigated whether treatment of mammalian cells with PTC containing this fusion protein induces toxic effects. Indeed, cell rounding confirmed successful injection of the fusion protein. Therefore, we concluded that affibodies principally can be injected by the system.

Based on the example of Z3-Raf, we further showed that also trimeric affibodies can be functionally injected by PTC into mammalian cells. The amount of affibodies within the cytosol was sufficient to block the important oncogenic Ras–Raf signaling pathway downstream of the EGF-receptor. The trimeric Raf affibody stably inhibited EGF-induced phosphorylation of ERK following delivery by PTC. Moreover, injection of Z3-Raf into HeLa, Panc-1, or Capan-2 cells significantly reduced cell proliferation indicated by less surface area covered by cells in colony formation assays. Panc-1 and Capan-2 cells are pancreatic cancer cells expressing an oncogenic Ras mutation [45,48]. The Z3-Raf-affibody was directed against the conserved Ras-binding domain of Raf variants [41]. Injection of this affibody as homotrimer seems sufficient to reduce oncogenic Ras-Raf signaling.

The A component of PTC may be engineered for selective cell binding. However, structural analysis suggest that there are several receptor binding domains on the surface of the shell [49]. Receptors identified so far include glycans and glycosylated proteins like Visgun on the surface of insect cells [50,51,52]. Nevertheless, with ongoing analysis and proper engineering of PTC there is a fair possibility to eventually manipulate specific cells in tissues or in living organisms.

Regardless of the strategy used, the fate of the intracellular proteins is not clear. Peptides or affibodies within the cell may be even more stable compared to the extracellular environment, because main protein degradation is restricted to membrane-enclosed lysosomes or to cytosolic proteasomes. Protein degradation within the cytosol therefore requires labeling by ubiquitination for degradation by the proteasome. In our studies, significant inhibition of colony formation points to a long-lasting effect of the injected Raf affibody. However, further studies are required for an exact analysis of the intracellular fate of the affibodies.

This work is a proof of principle that trimeric affibodies can be functionally injected by PTC into mammalian cells to influence signaling pathways.

## 4. Materials and Methods

### 4.1. Cell Culture

All cells were maintained at a constant temperature of 37 °C with 5% CO_2_. HeLa and Panc-1 cells were cultured in Dulbecco’s Modified Eagle Medium (DMEM) supplemented with 10% fetal calf serum (Anprotec, Bruckberg, Germany), 1% penicillin-streptomycin (Anprotec, Germany), and 1% non-essential amino acids (Thermo Fisher Scientific, Waltham, MA, USA). Cells were passaged every 2 to 3 days.

### 4.2. Cloning of Z3-Affibody Constructs

The Z3-affibodies were designed using theoretical protein engineering to meet the established criteria for PTC cargo [19]. The amino acid sequences of Zwt and ZRaf322 affibodies [40] served as templates. The complete construct included an N-terminal lysine leader sequence with nine lysine residues, followed by three affibodies oriented head-to-tail and connected by a linker. At the C-terminus, an Avi-tag was incorporated for site-specific biotinylation. Codon optimized double-stranded DNA fragments (IDTDNA, Leuven, Belgium) were cloned into the target vector backbones pGEX-4T1, pET-28a, and pET-28a-BC3 and transformed into *E. coli* TG1 cells. Construct integrity was confirmed by Sanger sequencing (Eurofins Genomics, Ebersberg, Germany).

### 4.3. Purification of Recombinant TcA and BC-Z3-Affibodies

pET-28a constructs were transformed into *E. coli* BL21 (DE3) cells. Cells were grown in LB medium supplemented with kanamycin, and protein synthesis induced with 25 µM Isopropyl-β-D-thiogalactopyranoside (IPTG). The bacterial cultures were incubated overnight (ON) at 28 °C with shaking. The next day, cultures were harvested by centrifugation (6000 rpm (Thermo Scientific F10-6—8593× *g*), 20 min, 4 °C). The bacterial pellets were resuspended in lysis buffer (300 mM NaCl, 20 mM Tris-HCl, 1 mM DTT, 500 µM EDTA, 10% (*v*/*v*) glycerol, pH 8.0) with the addition of 5 µg/mL DNase, 1 mg/mL lysozyme, and a complete protease inhibitor cocktail (Roche, Basel, Switzerland). Cell lysis was achieved through sonication, followed by centrifugation at 15,000 rpm (Sorval SS34—35,856× *g*), for 45 min at 4 °C. The resulting supernatant was incubated with Protino Ni-Nitrilotriacetic acid (Ni-NTA) beads. The resin was washed with wash buffer (500 mM NaCl, 20 mM Tris-HCl, 0.05% (*v*/*v*) Tween-20, 20 mM imidazole, 5% (*v*/*v*) glycerol, pH 8.0). Bound proteins were eluted by incubating the beads with elution buffer (500 mM NaCl, 20 mM Tris-HCl, 0.05% (*v*/*v*) Tween-20, 500 mM imidazole, 5% (*v*/*v*) glycerol, pH 8.0). The final protein solution was dialyzed overnight in dialysis buffer (100 mM NaCl, 50 mM Tris-HCl, 0.05% (*v*/*v*) Tween-20, 5% (*v*/*v*) glycerol, pH 8.0). Samples were concentrated using Vivaspin^®^ 6 Centrifugal Concentrators (10,000 MWCO; Sartorius, Göttingen, Germany) according to the manufacturer’s instructions. The concentrated eluates were further purified using anion exchange chromatography on a Mono Q 5/50 GL column (Cytiva, Marlborough, MA, USA) with an ÄKTApurifier UPC 100 system (Cytiva), followed by size exclusion chromatography on a Superdex™ 200 Increase 10/300 GL column (Cytiva). The purified protein was then concentrated, flash-frozen in liquid nitrogen, and stored at −80 °C until further use.

### 4.4. Purification of Z3-Affibodies

pGEX-4T1-Z3-affibody constructs were transformed into *E. coli* BL21 (DE3) cells. Cells were grown in LB medium supplemented with ampicillin to an OD_600_ of 0.6, and protein synthesis induced with 400 µM Isopropyl-β-D-thiogalactopyranoside (IPTG). The bacterial cultures were incubated overnight at 21 °C with shaking. The next day, cultures were harvested by centrifugation (6000 rpm (Thermo Scientific F10-6—8593× *g*), 20 min, 4 °C). The bacterial pellets were resuspended in lysis buffer (50 mM Tris/HCl, 50 mM NaCl, 2 mM PMSF, 10 mg/mL DNase, pH 7.5). Cell lysis was performed by sonication. Samples were centrifugation at 15,000 rpm (Sorval SS34—35,856× *g*) for 45 min at 4 °C. The supernatant was incubated with Glutathione Sepharose™ 4B resin (Cytiva). The resin was washed with wash buffer (50 mM Tris/HCl, 50 mM NaCl, pH 7.5), and bound proteins were eluted either using glutathione elution buffer (50 mM Tris/HCl, 50 mM NaCl, 10 mM glutathione, pH 7.5) or via thrombin cleavage (PBS, 2 mM CaCl_2_, 2 units/mL thrombin, pH 7.5). The protein solutions were dialyzed overnight in PBS, then concentrated using Vivaspin^®^ 6 Centrifugal Concentrators (10,000 MWCO; Sartorius, Germany) according to the manufacturer’s instructions. To remove thrombin, the concentrated affibodies were further purified using a Superdex™ 75 Increase column (Cytiva) on an ÄKTApurifier UPC 100 system (Cytiva). The purified protein was then concentrated, flash-frozen in liquid nitrogen, and stored at −80 °C until further use.

### 4.5. Surface Plasmon Resonance Measurements

Surface plasmon resonance (SPR) measurements were conducted using a Biacore X100 instrument (Cytiva, MA, USA). Purified Z3-wt and Z3-Raf affibodies were diluted in 10 mM sodium acetate buffer (pH 4.5) to a concentration of 25 µg/mL and subsequently immobilized on carboxymethylated dextran CM5 sensor chips (Cytiva, MA, USA), targeting immobilization levels of 1000–1500 resonance units (RU). Interaction candidates were injected at a flow rate of 30 µL/min at concentrations ranging from 11 to 900 nM. The association phase was set to 180 s, followed by a dissociation phase of 360 s. Measurements were performed in HBS-EP running buffer (10 mM HEPES, 150 mM NaCl, 3 mM EDTA, 0.005% Tween-20, pH 7.5). For C-Raf Ras-binding domain (RBD) measurements, 5 mM ZnCl_2_ was added. After each measurement cycle, binding was regenerated by injecting 10 mM HCl and 0.02% SDS for 60 s, followed by 10 mM NaOH for 60 s, and then a stabilization period of 300 s. The measured binding levels, recorded in resonance units (RU), were plotted using GraphPad Prism V5.00 (GraphPad Software, USA). Kinetic constants were determined with the Biacore X100 Evaluation Software V2.0.2 (Cytiva, MA, USA) based on a 1:1 binding model. Additionally, binding was assessed at equilibrium, 10 s before the end of the association phase for each concentration. Data were plotted as a dose–response curve, with regression analysis performed using GraphPad Prism V5.00.

### 4.6. Pull-Down with Magnetic Beads

The purified Z3-wt and Z3-Raf affibodies were covalently coupled to Pierce™ NHS-activated magnetic beads (Thermo Scientific, MA, USA) following the manufacturer’s protocol and stored at 4 °C until use. Z3-affibody-conjugated magnetic beads were equilibrated by washing three times with PBS containing 0.05% Tween 20 and 300 µM ZnCl_2_ (PBS-T-Zn). To pull down B-Raf, *E. coli* BL21(DE3) cells expressing recombinant B-Raf were resuspended in PBS-T-Zn supplemented with 2 mM PMSF and 10 µg/mL DNase. Cells were lysed by sonication, and debris was removed by centrifugation at 20,000× *g* for 30 min at 4 °C. The supernatant was diluted 1:10 with PBS-T-Zn and incubated with equilibrated Z3-affibody magnetic beads for 60 min at 4 °C on a rotary shaker. After incubation, the beads were separated using a magnetic stand, and the supernatant was discarded. The beads were then washed three times with PBS-T-Zn for 10 min at 4 °C. Bound proteins were eluted using Laemmli buffer and analyzed by SDS-PAGE and Western blotting. B-Raf proteins were detected using the BRAF antibody F-7 (Santa Cruz Biotechnology, Dallas, TX, USA) according to the manufacturer’s instructions. Protein bands were visualized with SuperSignal™ West Femto Maximum Sensitivity Substrate (Thermo Scientific, Waltham, MA, USA) and imaged. Band intensities were quantified using Multi Gauge V3.0 software (Fujifilm, Tokyo, Japan).

### 4.7. Pull-Down with GST-Glutathione Matrix

The GST-tagged C-Raf Ras-binding domain (RBD) was expressed in *E. coli* BL21(DE3) and coupled to Glutathione Sepharose 4B resin (Cytiva, Marlborough, MA, USA), as previously described. The RBD-GST-conjugated Glutathione Sepharose matrix was equilibrated three times with PBS-Zn (PBS + 500 µM ZnCl_2_). BL21(DE3) *E. coli* cells expressing recombinant Z3-wt or Z3-Raf affibodies were resuspended in PBS-Zn supplemented with 2 mM PMSF and 10 µg/mL DNase. Cells were lysed by sonication, and debris was removed by centrifugation at 20,000× *g* for 30 min at 4 °C. The resulting supernatant was diluted 1:10 with PBS-Zn and incubated with equilibrated Glutathione Sepharose matrix for 60 min at 4 °C on a rotary shaker. The solid phase was then pelleted by centrifugation at 1000× *g* for 5 min at 4 °C, and the supernatant was discarded. The matrix was washed three times with PBS-Zn, and bound proteins were eluted using Laemmli buffer. Elution fractions were analyzed by SDS-PAGE, followed by Coomassie Brilliant Blue staining of polyacrylamide gels. Gel images were taken, and affibody band intensities were quantified using Multi Gauge V3.0 software (Fujifilm, Tokyo, Japan).

### 4.8. In Vitro Injection Assays

Purified TcA and BC-Z3-wt proteins, stored at −80 °C, were thawed on ice prior to use. For each sample, 50 pM TcA was mixed with 10 pM BC-Z3-wt to achieve a 5:1 stoichiometric ratio of the TcA and BC components. As a control, 10 pM BC-Z3-wt was combined with an equivalent volume of protein storage buffer. The mixtures were incubated on ice for 1 h to allow for the assembly of PTC-Z3-wt holotoxin.

Microcon^®^ spin tubes (10,000 MWCO, Millipore, Burlington, MA, USA) were prepared with 100 µL of either pH 11 injection buffer (150 mM KCl, 20 mM CAPS/KOH, pH 11) or pH 7.5 injection buffer (150 mM KCl, 20 mM Tris/HCl, pH 7.5). The PTC3-Z3-wtholotoxins or BC3-Z3-wt proteins were added to the respective pH 7.5 or pH 11 injection buffers in the prepared spin tubes and incubated for either 10 or 60 min at room temperature. Following incubation, injection was terminated by neutralizing the pH 11 injection buffer with a predetermined volume of 200 mM HCl. Neutralization was confirmed with pH indicator paper (Carl Roth, Karlsruhe, Germany). Sample volumes were then reduced to approximately 20 µL by centrifugation (12,000× *g*, 30 min, 4 °C).

Injected Z3-wt affibodies were labeled through biotinylation of the C-terminally attached Avi-tag. Biotinylation was performed using the BirA500-RT BirA biotin-protein ligase lyophilized reaction kit (Avidity, San Diego, CA, USA) according to the manufacturer’s instructions. The 100 µL BirA biotinylation reaction was conducted for 2 h at room temperature and volumes were subsequently reduced to approximately 20 µL by centrifugation (12,000× *g*, 30 min, 4 °C). The reaction was halted by the addition of 5 µL of 5× Laemmli buffer. The samples were then subjected to SDS-PAGE, followed by Western blot analysis. Biotinylated proteins were detected using Strep-Tactin^®^ HRP (Iba Lifesciences, Göttingen, Germany) as per the manufacturer’s protocol. Protein bands were visualized using the SuperSignal™ West Femto Maximum Sensitivity Substrate (Thermo Scientific, Waltham, MA, USA) and imaged. Quantification of band intensities was performed using Multi Gauge V 3.0 software (Fujifilm, Tokyo, Japan).

### 4.9. Colony Forming Assays

HeLa, Panc-1, and Capan-2 cells were seeded into 6 cm dishes and cultured to ~80% confluency. Cells were then treated with 2 µg/mL TcA and 1 µg/mL of either BC-Z3-wt or BC-Z3-Raf for 1 h at 37 °C. To trigger the injection via surface-bound PTC, the culture medium pH was lowered by adding 80 µL of 1 M MES per 1 mL of medium, followed by a 5 min incubation at 37 °C. After treatment, cells were washed with PBS and detached using Trypsin/EDTA in PBS. Viable cell counts were determined for each treatment group, and equal numbers of cells were re-seeded into 35 mm six-well plates for a 10-day culture period. After the growth period, the medium was removed, and cells were washed with PBS, then fixed and stained with 0.5% crystal violet and 6% glutaraldehyde in PBS for 30 min. Stained cells were rinsed three times with deionized water (ddH_2_O). Well images were analyzed using ImageJ software V1.54p (NIH, Bethesda, MD, USA) to quantify the total area covered by cells in each dish. Each experimental day included three independent biological replicates per condition, corresponding to separate wells in different 6-well plates, for a total of nine replicates per condition (N = 9). To control for day-to-day and passage-to-passage variability, values were normalized to the mean of the three TcA/BC-Z3-wt measurements from the same day. The normalized values were subsequently plotted for each experimental group.

### 4.10. AP-1 Luciferase Reporter Assays

HeLa and Panc-1 cells were cultured to approximately 80% confluence and transiently transfected with pcDNA3.1 plasmids encoding Z3-wt or Z3-Raf under CMV promoter control. Each sample was co-transfected with the pAP-1-Luc plasmid, containing an AP1-regulated firefly luciferase (FF) reporter, and the pRL plasmid, encoding Renilla luciferase (RL) under the control of a constitutively active CMV promoter. Following transfection, cells were cultured in DMEM supplemented with 10% (*v*/*v*) fetal calf serum (FCS), 1% penicillin-streptomycin, and 1% non-essential amino acids for 16 h. Cells were then washed three times with PBS. Firefly and Renilla luciferase activities were quantified using the Dual-Glo^®^ Luciferase Assay System (Promega, Fitchburg, WI, USA) according to the manufacturer’s instructions. FF/RL ratios were calculated and plotted for each sample group. Per experimental day, three biological replicates per condition (Z3-wt and Z3-Raf) were performed in HeLa cells, and two in PANC-1 cells. Each replicate corresponded to an independently transfected culture plate with the respective pcDNA3.1 plasmid, AP1-luciferase reporter, and constitutively active Renilla reporter. Firefly (FF) and Renilla (RE) activities were measured after stimulation, with technical triplicates for HeLa and duplicates for PANC-1 averaged prior to analysis. FF/RE values were normalized to the mean of Z3-wt biological replicates from the same day to control for variability across days and passages. The resulting normalized FF/RE ratios were then plotted for each experimental group.

### 4.11. EGF Stimulation Assays

HeLa cells were grown to approximately 80% confluence in six-well plates and serum-starved overnight. Cells were then treated with 2 µg/mL TcA and 1 µg/mL of either BC-Z3-wt or BC-Z3-Raf for 2 h at 37 °C. Cells were exposed to 20 ng/mL epidermal growth factor (EGF) for 10 min. EGF stimulation was halted by removing the culture medium and immediately lysing the cells on ice. The samples were subjected to SDS-PAGE followed by Western blot analysis. Phosphorylated ERK (pERK) and total ERK levels were detected using the p44/42 MAPK (Erk1/2) (137F5) Rabbit Antibody (#4695) and the Phospho-p44/42 MAPK (Erk1/2) (Thr202/Tyr204) Antibody (#9101), both from Cell Signaling Technology (MA, USA). The SuperSignal™ West Femto Maximum Sensitivity Substrate (Thermo Scientific, MA, USA) was used for protein band visualization. Band intensities were quantified using Multi Gauge V 3.0 software (Fujifilm, Tokyo, Japan). To control for variation in gel loading and blotting efficiency across experiments, Na^+^/K^+^-ATPase was detected on the same PVDF membrane used for each ERK and pERK blot, using the Anti-alpha 1 Sodium Potassium ATPase antibody [464.6] (ab7671, Abcam, Berlin, Germany). Protein bands were visualized with SuperSignal™ West Femto Maximum Sensitivity Substrate (Thermo Scientific, Waltham, MA, USA) and quantified using Multi Gauge V 3.0 software (Fujifilm, Tokyo, Japan). On each experimental day, one independent biological replicate was performed per condition, with three total experiments for HeLa cells and five for PANC-1 cells. pERK and ERK band intensities were normalized to the TcdA/BC-Z3-wt + 10 min EGF stimulation condition from the same day, then adjusted for gel loading and blotting efficiency using Na^+^/K^+^-ATPase signals (0 min EGF set to 1). pERK/ERK ratios were calculated for each sample. To standardize presentation, values were finally scaled so that the mean of the TcdA/BC-Z3-wt + 10 min EGF condition across all experiments equaled 1.

### 4.12. Statistics

Statistical analyses were performed using GraphPad Prism version 5.00 for Windows (GraphPad Software, Boston, MA, USA). Unless otherwise specified, data are presented as the mean ± standard deviation (SD). *p*-values less than 0.05 were considered statistically significant and are indicated as follows: n.s., not significant; * *p* < 0.05; ** *p* < 0.01; *** *p* < 0.001; **** *p* < 0.0001. We acknowledge advice from the Institute for Medical Biometry and Statistics at the University of Freiburg.

## Data Availability

The original contributions presented in this study are included in this article and Supplementary Materials. Further inquiries can be directed to the corresponding author.

## Supplementary Materials

The following supporting information can be downloaded at: https://www.mdpi.com/article/10.3390/toxins17090448/s1, Figure S1: Organization of Z3 affibodies; Figure S2: Colony formation assay of Capan-2 cells.
