# Supplementary material for: Injection of Affibodies by a Self-Organizing Bacterial Syringe to Interfere with Intracellular Signaling
Source: Toxins (Basel). 2025 Sep 5;17(9):448. doi: 10.3390/toxins17090448 (PMC12474113; doi:10.3390/toxins17090448)
Supplement: Supplementary file 1 [file toxins-17-00448-s001.zip › toxins-3847663-supplementary.pdf]

Supplements

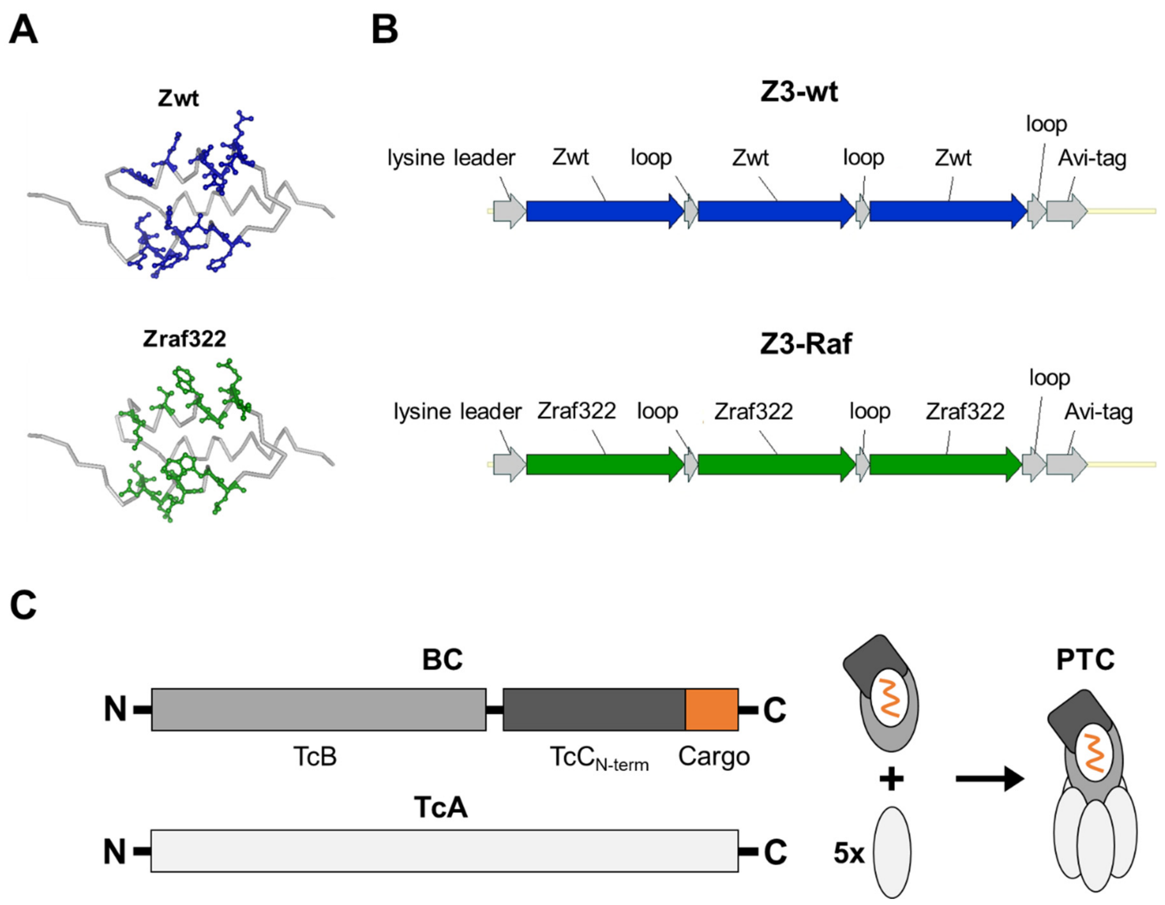

Figure S1. (**A**) Predicted 3D structure of the wild-type protein A Z-domain (Zwt, blue) and the monomeric Zraf322 (green) affibody. The three-helix bundle backbone is shown in grey. The 13 mutated amino acids are highlighted using a ball-and-stick model, with distinct colors for each affibody molecule. Structural predictions were generated using the Phyre2 protein structure prediction tool. (**B**) Schematic representation of Z3-affibodies: Z3-wt (blue) and Z3-Raf (green). Each Z3 construct includes an N-terminal lysine leader, followed by three affibody monomers (Zwt, Zraf) linked by short loops and an AVI-tag for detection. (**C**) Schematic representation of PTC assembly. TcB and TcC are co-expressed as a BC fusion construct, where a foreign cargo molecule replaces the wild-type hvr at the C-terminus. TcA is expressed separately. Five TcA subunits associate with the BC protomer to form the PTC holotoxin.

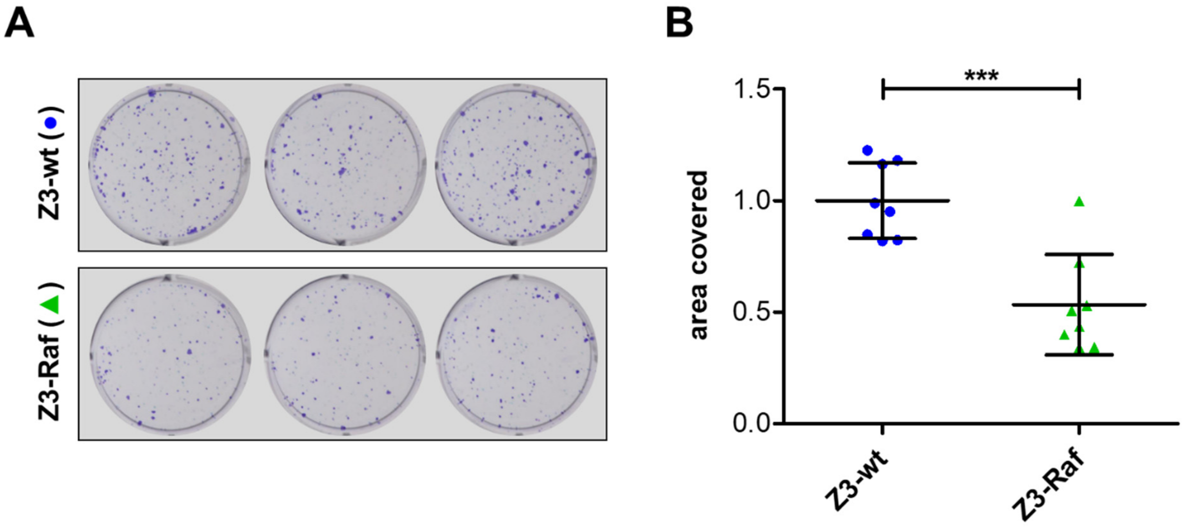

Figure S2. Colony formation assay of Capan-2 cells treated with PTC3 loaded with Z3 affibodies as cargo. (**A**) Representative images of stained cell colonies after 10 days of growth in 35 mm dishes. Cells were seeded at equal densities and pretreated for 2 hours with PTC3 containing Z3-wt, Z3-Ras, or Z3-Raf affibodies prior to the growth phase. (**B**) Quantification of colony formation in Capan-2 cells. Data are presented as the relative area covered by cell colonies for each group. For each experimental day, all values were normalized to the mean of the PTC-Z3-wt–treated group, which was set to 1. Mean ± standard deviation from eight independent experiments (N = 8) are shown. Statistical significance was determined using Student’s t-test (***, $p < 0.001$).
